# Bioprinting for bone tissue engineering

**DOI:** 10.3389/fbioe.2022.1036375

**Published:** 2022-11-25

**Authors:** Xin Kang, Xiao-Bo Zhang, Xi-Dan Gao, Ding-Jun Hao, Tao Li, Zheng-Wei Xu

**Affiliations:** ^1^ Department of Spine Surgery, Honghui Hospital, Xi’an Jiao Tong University, Xian, Shaanxi, China; ^2^ Department of Orthopedics, Lanzhou University Second Hospital, Lanzhou, Gansu, China

**Keywords:** four-dimensional bioprinting, stimulus-responsive, bone tissue engineering, challenges, review

## Abstract

The shape transformation characteristics of four-dimensional (4D)-printed bone structures can meet the individual bone regeneration needs, while their structure can be programmed to cross-link or reassemble by stimulating responsive materials. At the same time, it can be used to design vascularized bone structures that help establish a bionic microenvironment, thus influencing cellular behavior and enhancing stem cell differentiation in the postprinting phase. These developments significantly improve conventional three-dimensional (3D)-printed bone structures with enhanced functional adaptability, providing theoretical support to fabricate bone structures to adapt to defective areas dynamically. The printing inks used are stimulus-responsive materials that enable spatiotemporal distribution, maintenance of bioactivity and cellular release for bone, vascular and neural tissue regeneration. This paper discusses the limitations of current bone defect therapies, 4D printing materials used to stimulate bone tissue engineering (e.g., hydrogels), the printing process, the printing classification and their value for clinical applications. We focus on summarizing the technical challenges faced to provide novel therapeutic implications for bone defect repair.

## 1 Introduction

Globally, many reconstructive procedures are required yearly to cope with bone defects caused by accidental fractures, osteoporosis, cancer, and hereditary diseases such as chondromalacia (Sun et al., 2022). Although bone has the intrinsic property of self-repair, in many cases, bone cannot fully regenerate and requires external stimulation (Chircov et al., 2021; Park et al., 2022), which leads to a very high number of patients with bone defects requiring bone grafts or replacements. Current solutions to address bone defect disorders include medical procedures, grafting, and pharmacological treatments. Nevertheless, such approaches are inefficient and interrupt bone regeneration and microbial contamination (Chircov et al., 2021). There is a need to explore alternative techniques to treat bone defects due to the disadvantages of donor site morbidity and donor shortage associated with autologous and allogeneic grafts. The rapid development of three-dimensional (3D) stents for *ex vivo* implants in tissue engineering for tissue repair and stem cell transplantation holds promise for meeting the demand for bone defects. 3D bioprinting technology can be used to produce patient-specific structures in a time-saving and cost-effective manner, resulting in greater flexibility in material selection and more biocompatible structures than metal implants produced through traditional manufacturing methods (Li et al., 2022). Currently, 3D bioprinting technology has been used to manufacture different human tissues (e.g., bone and blood vessels) and extracellular matrices (Yu et al., 2019). Past research in 3D bioprinting technology has focused on incorporating regenerative bone structures such as cells or bioactive factors. Most of the materials used in the study are some engineered hydrogel materials, because bioprinting most importantly requires bioinks with deformable capabilities, but also reduced immunogenicity and some ability to release targeted drugs. Based on the loading function of bioinks, the induction of differentiation of bone marrow mesenchymal stem cells into myeloid cells by specific life active molecules such as connective tissue growth factor (CTGF) and transforming growth factor-β3 (TGF-β3) loaded into specific biomaterials, along with printed IVD structures with good biomechanical function (Sun et al., 2021), might be effective for the treatment of degenerative intervertebral disc changes. In addition, 3D bioprinted material scaffolds can accommodate the controlled release of bioactive molecules (Matai et al., 2020). Process parameters affecting bioprinting and bioink biomaterials are gradually gaining consensus, and bioprinted skin, heart, bone, cartilage, liver, lung, nerve, and pancreatic tissues are gradually being used in clinical applications (Matai et al., 2020). Of course, the availability of bioinks is also influenced by the concentration of each hydrogel component, e.g. GelMA concentration can regulate the formation of capillary structures (Anada et al., 2019).

However, for further clinical applications, issues such as reconstructing irregular and personalized bone tissue, vascularization of regenerated bone and nerve regeneration, and characterization of mechanically 3D printed structures still need to be addressed (Ashammakhi et al., 2019; Qasim et al., 2019).

For these reasons, four-dimensional (4D) printing introduces time thinking, including but not limited to innovative materials and biomedical research (Wan et al., 2020). 4D-printed structures can be gradually changed under different stimuli to adapt to personalized needs (Zhang et al., 2019a). It should be noted that the structure or function of the 4D-printed structure remained stable. In contrast, a 3D manufactured structure is the vehicle for shape or function transformation, and controlled degradation is not part of 4D printing. 4D bioprinting has been a hot topic of research in recent years, and the bioink used is a controllable hydrogel material that changes over time to meet the structural or functional needs of the organism. Currently, bioprinting research in orthopedics is focused on the osteogenic transformation of mesenchymal stem cells (MSCs), and bioprinting would allow the generation of hydrogel scaffolds with a high degree of spatial control over MSCs distribution within the bioprinted structures. Mixing of fibrin and collagen and their respective hydroxyapatite may improve MSC’s survival (Benning et al., 2017). Byambaa et al. (2017) developed cell-filled cylindrical elements made of GelMA hydrogels containing silicate nanoplates to induce osteogenesis and synthesized hydrogel preparations with chemically conjugated vascular endothelial growth factor to promote vascular spreading. Many previous studies have reported successful osteogenic differentiation of hydrogel-loaded bone mesenchymal stem cells (BMSCs), and we do not list them all here. This paper intends to provide some new insights into the clinical treatment of orthopedics by summarizing the more widely researched bioprinting technologies while offering help to faster and better application of bioprinting in orthopedic treatment.

## 2 Stimulus-responsive materials

The shape and function of printed structures help maintain biosynthetic processes and self-renewal. These structures undergo dynamic conformational shifts over time to adapt to the needs of the surrounding microenvironment. Stimulus-responsive biomaterials provide a finer strategy for shape transformation (Anada et al., 2019). Shape-memory materials have been found to have the ability to fix temporary shapes and change them into permanent structures in response to appropriate stimuli.

**FIGURE 1:**
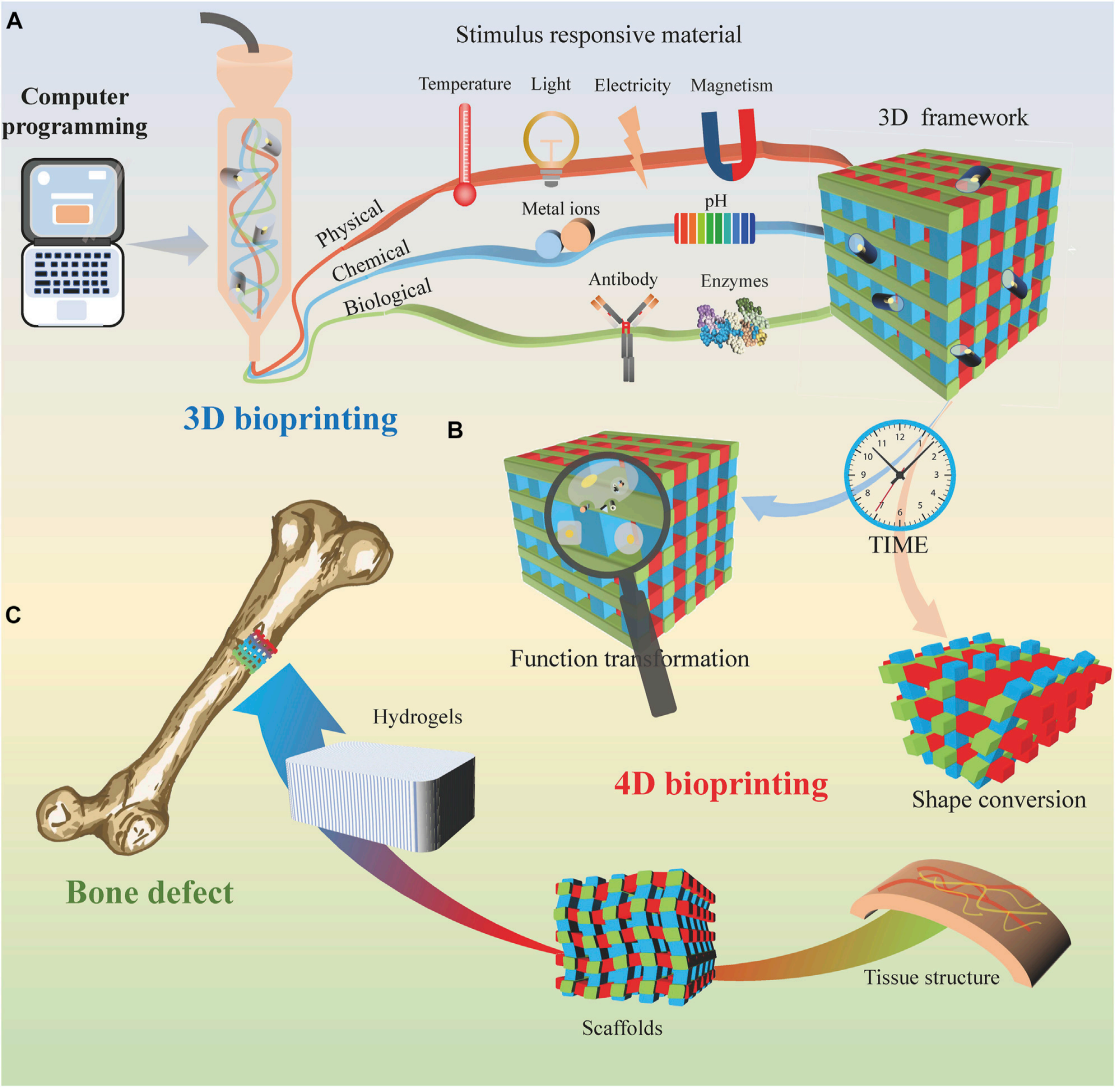
4D bioprinting for bone tissue engineering. **(A)** 3D bioprinting process based on different stimulus-responsive biomaterials. **(B)** With time, the 3D printing bracket transformed into a 4D printing organizational structure. **(C)** 4D bioprinted tissue is used to cure bone defects.

Hydrogels are promising adaptive materials due to their excellent biocompatibility (He et al., 2021). 4D biomanufacturing using deformed hydrogels to form pre-designed hydrogel biostructures containing 3D cells. It has the potential to achieve precise mimicry of the function and complex structure of natural tissues or organs. Due to physical or chemical cross-linking properties, shape memory hydrogels have been developed. They can be self-assembled by achieving a reversible state transformation and function as needed (Neffe et al., 2021). Stimulus-responsive materials are required to fabricate printed structures in response to external stimuli for shape transformation and functional tuning (Park et al., 2022). According to different types of stimuli, smart materials are divided into physical, chemical, and biological stimulus responsiveness (Figure 1A).

### 2.1 Physical stimulation

#### 2.1.1 Temperature

Physical stimulation mainly includes temperature, light, electricity, and magnetic field (Figure 1A). Wu et al. (Wu et al., 2021) presented an approach to imitating dynamic movements using hydroxybutyl methacrylated chitosan (HBC-MA). When the ambient temperature increases, additional thermal cross-linking of the photocross-linked HBC-MA hydrogels occurs. Previously reported that lotus root gel could be mixed with pigments in the right ratio and printed in 4D at the right temperature. (Chen et al., 2021). The thermal cross-linking process not only forms extra pores within the hydrogel but also involves water demineralization. On the other hand, when the ambient temperature decreases, the hydrophobic interactions are released, increasing the water swelling rate as the hydrophilic chains are revealed.

#### 2.1.2 Light

Directed cell growth in light-tunable materials can trigger changes in biomaterial properties. Various research breakthroughs have been achieved with advances in science and technology, including synthetic microvessels and induced cell differentiation, laying an essential foundation for cell culture. Photoresponsive hydrogels are a useful platform for simulating 4D changes in the ECM by integrating photoresponsive elements into a synthetic matrix and transforming them into mechanical stimuli. The main light currently used is UV light, but UV light itself is cytotoxic and has low tissue penetration. Zheng et al. (2020) embedded upconverting nanoparticles into hydrogels modified with light-activated cell adhesion motifs, resulting in upconverting nanoparticles converting near-infrared (NIR) to local UV emission and activating photochemical reactions as requested.

#### 2.1.3 Electricity

Most electrically responsive materials are polyelectrolyte polymers that expand, contract, or fold under the action of an external electric field. Electroresponsive hydrogels are electrically conductive material compositions that respond to electrical stimuli by reversibly absorbing and expelling water. Stimulus translation is achieved through water and action, elastic compliance, biocompatibility, and electrochemical efficacy. Electrically stimulated conductive hydrogels can control drug delivery and induce stem cell differentiation, vascular regeneration, and neurogenesis (Khan et al., 2022). In short, the electrical signal is applied in the microfluidic platform to use the conductive hydrogel as an effective actuator.

#### 2.1.4 Magnetism

Magnetism driven mobile micromachines have helped drive advances in minimally invasive targeted therapies. Still, their need to rely exclusively on magnetic programming to perform specific tasks may diminish their potential performance and functional versatility. Combining mobile magnetic minicomputers with stimulus-responsive materials may define new functions for their independent control. Magnetic hydrogels are hydrogel materials based on magnetic response; e.g., alginate hydrogels can mimic rotating magnets for pressure stimulation in gastric cancer (Zhao et al., 2021).

### 2.2 Chemical stimulation

Chemical stimuli mainly include pH and chemical ions (Figure 1A). These pH-responsive materials exhibit different shape transitions at critical pH values. Chen et al. (2021) investigated the 4D printing of a lotus root gel compounded with a pigment that responds to pH change and alters the color. Many previous studies have reported strategies to create scaffolds that support cellular structures by cross-linking with biological ions such as calcium ions (Ca^2+^) and intracellular free zinc (Zn^2+^). The concentration of Zn^2+^ release can be controlled for bioactive glass synthesis steps for bioprinted material systems (Guduric et al., 2021).

### 2.3 Biostimulation

Biostimulation consists mainly of active biomolecules, such as antibodies and enzymes (Figure 1A). Enzymes play a crucial role in many biochemical reactions process. The substrate of the enzyme should act as a functional side group or cross-linker of the hydrogel. Over time, the hydrogels can change from a random coil conformation to a β-folded conformation. This enzyme-sensitive hydrogel allows for rapid cross-linking, appropriately timed degradation, and demand-adapted morphological characterization.

## 3 Steps and classification of four-dimensional bioprinting

4D biology evolved from 3D bioprinting, so the printing steps are similar to 3D printing. Data acquisition is first performed, and 3D models are recreated by magnetic resonance imaging (MRI), computed tomography (CT), and other techniques. Next, select the printing materials, including growth factors, cells, *etc*. They are usually called bioinks, with the most commonly used hydrogel-loaded cells. Again, the printing parameters are set, and bioprinting is performed. Finally, printed tissues or organs are used for functional applications in the human body.

Similarly, 4D printing methods are similar to 3D printing methods. Depending on the printing method, it can be divided into extrusion bioprinting, droplet bioprinting, light-curing bioprinting, *etc.* (Gu et al., 2020). The extrusion technology has wide range of biocompatible materials that can be printed (e.g., cell-loaded hydrogels), but the accuracy is generally limited (Duan et al., 2013). The bioinks also require gelation or curing, and shear forces are not negligible for cell damage. Droplet bioprinting offers the advantages of being inexpensive, having high accuracy and speed, and compatibility with various biological materials. It can print different cells, biomaterials or growth factors simultaneously and has a fast printing speed. However, it is impossible to print high concentrations of cells, while low-viscosity materials reduce the structural strength. At the same time, inkjet printing causes greater mechanical or thermal damage to cells (Cui et al., 2010). Laser-assisted printing is contactless printing and avoids the problem of clogging nozzles in the extrusion or inkjet bioprinting of cells/biomaterials. Nevertheless, the printing cost is higher, and there is less printable material (Murphy and Atala, 2014). In conclusion, a more suitable printing method needs to be selected according to different needs.

## 4 Application in bone tissue engineering

### 4.1 Injectable stimuli-responsive hydrogels

4D printing strategies demonstrate the potential to fabricate biologically complex multilayered tissue structures, which show overwhelming advantages in the personalized repair of bone defects. Several injectable thermoresponsive polysaccharide hydrogels as carriers for different cellular or inorganic composites (Graham et al., 2019). These modified biomaterials (e.g., hydroxybutyl chitosan) exhibit suitable critical solution temperatures and can be converted to a gel state at body temperature. Adding mineral components can significantly improve hydrogels’ mechanical strength. Moreover, this injectable hybrid hydrogel has ideal rheological properties and *in vivo* self-coagulation ability to fill minor and irregularly shaped defects. Currently, bone morphogenic protein-2 nanoparticles (Aghajanpour et al., 2022) and notoginsenoside R1 (Wang, Yan, Lan, Wei, Zheng, Wu, Jaspers, Wu, Pathak) are incorporated into hydrogel systems as induction factors to enhance the MSCs’ differentiation ability to repair bone defects.

### 4.2 Shape memory scaffolds

Previous studies have demonstrated the ability of 3D printing to accurately control scaffold microstructure and composition at the microscopic scale, including organic scaffolds, inorganic scaffolds, and hybrid scaffolds; organic scaffolds include polymer-based systems and bioprinting, while inorganic scaffolds include ceramic scaffolds and metal scaffolds (Qu et al., 2021). 4D printing has also been investigated for printing structural scaffolds. 4D printed structures can be used as implants to repair bone defects, where the shape of the scaffold is transformed to fill the space after implantation (Zhang et al., 2019a).

### 4.3 Print smart materials

4D printing is an innovative manufacturing method that combines 3D printing with smart materials that can respond to different stimuli and adapt them to the surrounding microenvironment. In contrast to 3D printing, where printed structures can achieve shape changes after fabrication, bioinks have shape-denaturing properties. Various smart materials have been developed to fulfill their dimensional transformation based on specific external stimuli (e.g., temperature and light). Smart materials can be loaded with various mesenchymal stem cells or myoblasts for their final shape or functional transformation (Yang et al., 2019). For biomedical clinical applications, smart materials need to avoid *in vivo* responses such as inflammation and immunity and support normal physiological functions of the organism. Therefore, bio-ink materials should be sufficiently biocompatible, have a pre-designed strict shape or functional variation, and make physiologically active adaptations to the readily changing local microenvironment (Zheng et al., 2020).

### 4.4 Print micro organization

Regeneration of the microvasculature and nerves is one of the main challenges of bone transplantation. The neurovascular unit model constructed by printing technology facilitates the observation of neurovascular units and their positive role in monitoring and investigating the mechanisms of various central nervous system diseases (Dong et al., 2022). It has been reported that NIR can penetrate approximately 2.5 mm thickness of skin tissue and activate the angiogenic response. Human umbilical vein endothelial cells embedded in this hydrogel form a blood vessel network at a predefined geometry by the irradiation pattern (Zheng et al., 2020). The thrombotic inflammatory response observed *in vitro* through 3D-printed blood vessels (Figure 1B) contributes to understanding the pathophysiology of vascular diseases. It also provides a survey tool for clinicians to assess patients’ conditions and select therapeutic agents (Gold et al., 2021).

Furthermore, local and preprogrammed calcification and direct fibronectin can trigger biofilm formation during bioprinting by enzyme trapping in 4D hydrogels (Figure 1C). In addition, these stimulus-responsive cell carriers exhibit cell-homing properties and can be used as clinically applicable carriers for site-specific injury repair (Yun et al., 2018).

## 5 Challenges

Although stimulus-responsive biomaterials and hydrogel-based scaffolds have had significant success in bone regeneration, 4D bioprinting still has multiple challenges. First, the translation of existing stimulus-responsive biomaterials into optimized bioinks remains challenging (Anada et al., 2019). Biomanufacturing methods have been investigated for *in vivo* applicability. However, their direct application to bioinks may not be straightforward. The adverse effects of the printing procedure on cellular bioscaffolds and the potential for mass production remain low.

Second, current 4D printed structures’ deformation is still simple (e.g., folding) and cannot meet the complex needs in clinical applications. The generation or release of internal stresses also needs to be precisely controlled when the stimulus-responsive material is deformed. The stimulus responsiveness needs to be maintained over time without losing its unique properties. In addition, biomaterial scaffolds need to be developed with sufficient mechanical strength, as the mechanical properties of the printed structures will be significantly reduced after repeated folding/unfolding (Ionov, 2018).

Again, the interaction between the printed structure and the host microenvironment cannot be ignored. For example, extreme UV levels and pH changes may negatively affect cell viability. In contrast, suitable temperature and Ca^2+^ concentration changes do not seem to adversely affect living cells (Aghajanpour et al., 2022), and relatively mild 4D conversion stimulates the mechanism to be more friendly to the host environment. Despite substantial progress in hydrogel-based 4D biomanufacturing, available cytocompatible materials capable of 4D conversion in a physiological environment are still limited. The materials used must be strictly cytocompatible, and the hydrogels must be manufactured under mild conditions to ensure high cell viability (Miao et al., 2017). To meet these stringent requirements, cytocompatible stimuli, such as Ca^2+^, light irradiation, polysaccharides and peptides, can be introduced. However, using the extracellular matrix of human tissue to enhance ink may be an immune response (De Santis et al., 2021).

Finally, cellular activities within human tissues are susceptible to multiple stimuli, such as neuromodulation, humoral regulation, and self-regulation (Qasim et al., 2019). This requires printed biological structures to undergo complex intricate shape transformation and conversion processes before realizing their full functions. Based on this, computer design techniques and complex multiple stimulus-response procedures can be introduced to achieve this. However, there is still a need to weigh the pros and cons between the expensive cost of programmable biomaterials and the superiority of 4D bioprinted structures. Although current 4D bioprinting technologies appear to be available for clinical drug delivery (Tang et al., 2019) and cell therapy (Zhang et al., 2019b), most recent studies are limited to animal and cellular studies. Previously studied 4D materials are non-degradable and/or non-biocompatible, which in certain procedures perhaps limits their application in regenerative medicine. Lee et al. (2021) found that bioprinting of high-density cellular oxidized and methacrylated alginate and GelMA allowed the formation of more complex structures with defined 4D geometric variations, suggesting that further improvements and advances in printing parameters and techniques are possible. In conclusion, the above barriers are related to these techniques and angiogenesis, mechanical strength, sustainability of alternatives, vascular nerve regeneration, and clinical trials.

## 6 Conclusion

In summary, 4D bioprinting is emerging as a promising alternative therapy for tissue regeneration, using hydrogels as bioinks to print a variety of complex active, multilayered and functional bone tissues that meet functional needs. Despite the many technical challenges, the future of 4D bioprinting will continue to evolve toward cost-effective mechanical strength and functional bone construction.

## Abbreviations

4D, four-dimensional; 3D, three-dimensional; MSCs, mesenchymal stem cells; BMSCs, bone mesenchymal stem cells; HBC-MA, hydroxybutyl methacrylated chitosan; NIR, near-infrared; Ca^2+^, calcium ions; Zn^2+^, intracellular free zinc; CT, computed tomography; MRI, magnetic resonance imaging.
